# HIV infection is an independent risk factor for decreased 6-minute walk test distance

**DOI:** 10.1371/journal.pone.0212975

**Published:** 2019-04-24

**Authors:** Tom E. Robertson, Mehdi Nouraie, Shulin Qin, Kristina A. Crothers, Cathy J. Kessinger, Deborah McMahon, Divay Chandra, Lawrence A. Kingsley, Ruth M. Greenblatt, Laurence Huang, Meghan E. Fitzpatrick, Alison Morris

**Affiliations:** 1 Department of Medicine, Division of Pulmonary, Allergy and Critical Care Medicine, University of Pittsburgh School of Medicine, Pittsburgh, Pennsylvania, United States of America; 2 Department of Medicine, Division of Pulmonary, Critical Care and Sleep Medicine, University of Washington, Seattle, Washington, United States of America; 3 Department of Infectious disease and Microbiology, Graduate School of Public Health, University of Pittsburgh, Pittsburgh, Pennsylvania, United States of America; 4 Department of Clinical Pharmacy, University of California at San Francisco, San Francisco, California, United States of America; 5 Department of Medicine, Division of Pulmonary and Critical Care Medicine and HIV, Infectious Diseases and Global Medicine Division, University of California San Francisco, San Francisco, California, United States of America; 6 Department of Immunology, University of Pittsburgh School of Medicine, Pittsburgh, Pennsylvania, United States of America; Vanderbilt University Medical Center, UNITED STATES

## Abstract

**Background:**

Ambulatory function predicts morbidity and mortality and may be influenced by cardiopulmonary dysfunction. Persons living with HIV (PLWH) suffer from a high prevalence of cardiac and pulmonary comorbidities that may contribute to higher risk of ambulatory dysfunction as measured by 6-minute walk test distance (6-MWD). We investigated the effect of HIV on 6-MWD.

**Methods:**

PLWH and HIV-uninfected individuals were enrolled from 2 clinical centers and completed a 6-MWD, spirometry, diffusing capacity for carbon monoxide (DL_CO_) and St. George’s Respiratory Questionnaire (SGRQ). Results of 6-MWD were compared between PLWH and uninfected individuals after adjusting for confounders. Multivariable linear regression analysis was used to determine predictors of 6-MWD.

**Results:**

Mean 6-MWD in PLWH was 431 meters versus 462 in 130 HIV-uninfected individuals (p = 0.0001). Older age, lower forced expiratory volume (FEV_1_)% or lower forced vital capacity (FVC)%, and smoking were significant predictors of decreased 6-MWD in PLWH, but not HIV-uninfected individuals. Lower DL_CO_% and higher SGRQ were associated with lower 6-MWD in both groups. In a combined model, HIV status remained an independent predictor of decreased 6-MWD (Mean difference = -19.9 meters, p = 0.005).

**Conclusions:**

HIV infection was associated with decreased ambulatory function. Airflow limitation and impaired diffusion capacity can partially explain this effect. Subjective assessments of respiratory symptoms may identify individuals at risk for impaired physical function who may benefit from early intervention.

## Introduction

Half of persons living with HIV (PLWH) living in the United States are over 50 years old [1] and are increasingly affected by age-associated and chronic comorbid conditions [2, 3]. HIV infection is similarly associated with increased frailty, as well as declines in lung and cardiovascular health [4] that have potential impact on physical function. In one large study of United States male veterans, decline in scale-based self-reported physical function per year was greater in PLWH than HIV-uninfected individuals, and chronic pulmonary disease appeared to uniquely modify this effect [5]. While there have been a host of studies assessing chronic pulmonary disease and aberrant pulmonary physiology in PLWH [6–8], there has been limited research specifically assessing cardiopulmonary functional capacity, highlighting the lack of insight into the clinical determinants of declining physical function in this high-risk group [9]. Additionally, in heterogenous populations of PLWH, the relationships among demographic and clinical variables and measures of physical function are relatively unexplored [10].

Six-minute walk distance (6-MWD) is a validated metric for assessing functional aerobic exercise capacity. It is a predictor of mortality in the general population [9, 11] and in PLWH [9]. Studies in a predominantly male veteran population found that HIV was not an independent predictor of 6-MWD, but that forced expiratory volume in one second (FEV_1_) percent-predicted and radiographic emphysema were uniquely associated with decreased 6-MWD in PLWH [12, 13]. These findings suggest a link between the increased risk of obstructive pulmonary disease [6, 7] and diminished respiratory-related health status [14] that are observed in PLWH in epidemiologic studies, but conclusions are not generalizable due to the relatively homogenous cohort.

We assessed the independent effect of HIV on subjective and objective measures of cardiopulmonary function in a cohort of PLWH and HIV-uninfected individuals and identified associated clinical and demographic variables as well as potential biomarkers.

## Materials and methods

### Participants

Individuals with documented HIV infection as well as HIV-uninfected individuals who were 18 years of age or older were recruited from the University of Pittsburgh and University of California San Francisco as part of the Pittsburgh HIV Lung Research Cohort [8, 15]. Participants were recruited using posted advertisements and from the Multicenter AIDS Cohort Study (MACS) [16], the Women’s Interagency HIV Study (WIHS) [17], and the Pitt Center for Treatment research registry. Exclusion criteria were acute respiratory symptoms (new or increasing cough or shortness of breath) within the previous four weeks, prior thoracic surgery, or contraindication to either pulmonary function testing or 6-MWD testing. The University of Pittsburgh and University of California San Francisco IRBs approved the protocol, and all participants signed written informed consent.

### Data collection/methods

Standardized questionnaires were used to obtain demographic and baseline clinical data including smoking history and adherence to antiretroviral therapy (ART). Review of the electronic medical record or MACS and WIHS databases were used to obtain CD4+ T-lymphocyte count (CD4) and plasma HIV RNA levels within 6 months from current visit. Participants performed pre- and post-bronchodilator spirometry and measurement of the diffusing capacity for carbon monoxide (DL_CO_) per American Thoracic Society (ATS) and European Respiratory Society (ERS) standards [18–20]. Spirometry reference values were determined using the third National Health and Nutrition Examination Survey equations [21]. Equations by Neas et al. were used as reference values for DL_CO_ and DL_CO_ percent predicted was adjusted for hemoglobin and carboxyhemoglobin [22]. Only tests meeting ATS standards or deemed acceptable by a trained pulmonologist as acceptable were included in analysis [18, 21–24]. Respiratory symptoms were assessed by patient self-report of new or increasing cough or dyspnea. Assessments of respiratory symptoms were performed using a modified version of the St George’s Respiratory Questionnaire (SGRQ) and the Modified Medical Research Council (MMRC) questionnaire. SGRQ is a 50-item questionnaire that measures the domains of symptoms, activity and impact with s ranging from 0–100 with higher scores indicating worse functional impairment [25]. MMRC is a five-point scale based on degree of breathlessness generated by various physical activities [26]. All participants completed a six-minute walk distance (6-MWD) test according to the American Thoracic Society guidelines [18, 27]. Total distance walked was recorded per standard protocol. The reference range of the 6-MWD test is age- and gender-dependent with mean (SD) of 571 (90) meters in healthy adults [28].

### Statistical analysis

Demographic, clinical and laboratory markers as well as PFT measurements were compared between PLWH and HIV-uninfected groups using Student’s t test or Chi-squared test. Correlation coefficients between specific variables and 6-MWD (Pearson correlations) were calculated separately by HIV status. Additionally, we used a backward stepwise approach using predictors with P ≤ 0.2 from bivariate analyses (besides height as a known 6-MWD predictor) for multivariable linear regression models to determine predictors of 6-MWD in PLWH and HIV-uninfected participants. An interaction between each variable and HIV status was calculated to test predictors of 6-MWD in PLWH versus HIV-uninfected individuals. Each model was tested for linearity of relationship, distribution of residual, high influential observations, and interactions. A P value less than 0.05 was considered statistically significant. All analyses were performed in STATA 14.2 (StataCorp., College Station, TX).

## Results

### Characteristics of the study population

Two hundred ninety-seven PLWH and 130 HIV-uninfected participants were included (Table 1). Median age was 51 years, 40% were female, 51% were African-American. Median pack-year smoking in HIV-infected individuals was 7.8 vs. 5.5 in HIV-uninfected individuals (p = 0.06). Most HIV-infected participants were receiving ART and 65% had undetectable plasma HIV RNA levels. Median CD4 cell counts were generally within normal range (median CD4 cells/μL, 618; IQR 434–856).

**Table 1:** Distribution of demographic and clinical variables by HIV status.

	All (n = 427)	HIV-uninfected (n = 130)	PLWH (n = 297)	P value
Age, median years (IQR)	51 (44–57)	51 (43–57)	52 (45–56)	0.94
Female, % (n)	39.6 (165)	46.2 (60)	35.4 (105)	0.035
Race, % (n)				
Caucasian	41.5 (177)	46.2 (60)	39.4 (117)	0.32
African-American	51.3 (219)	48.5 (63)	52.5 (156)	
Other	7.2 (31)	5.3 (7)	8.1 (24)	
Site, % (n)				
Pittsburgh	73.3 (313)	74.6 (97)	72.7 (216)	0.69
University of California San Francisco	26.7 (114)	25.4 (33)	27.3 (81)	
Height cm, median (IQR)	173 (165–180)	170 (163–178)	173 (165–180)	0.07
Weight kg, median (IQR)	78 (70–91)	82 (73–95)	78 (69–90)	0.06
Smoking status, % (n)				
Never	30.9 (132)	36.2 (47)	28.6 (85)	0.27
Current	44.3 (189)	42.3 (55)	45.1 (134)	
Former	24.8 (106)	21.5 (28)	26.3 (78)	
Pack-year, median (IQR)*	15.3 (6.3–31.2)	13.9 (5.7–26.3)	16.1 (6.9–32.3)	0.26
Illicit drug usage, % (n)	88.3 (377)	86.2 (112)	89.2 (265)	0.36
Hemoglobin g/dL, median (IQR)	13.8 (12.7–14.8)	14.2 (12.9–15.0)	13.7 (12.7–14.7)	0.11
VL, median copies/ml (IQR)^1^		--	39 (19–63)	--
VL undetectable, % (n)^1^		--	64.5 (182)	--
CD4 cell count/μL, median (IQR)^2^		--	618 (434–856)	--
Current ART, % (n)^3^		--	92.6 (275)	--
SBP mmHg, median (IQR)	125 (115–138)	127 (118–139)	124 (114–137)	0.07
DBP mmHg, median (IQR)	79 (72–87)	81 (73–89)	79 (72–86)	0.38
FEV_1_% post-BD, median (IQR)^4^	92 (79–104)	93 (80–103)	92 (77–105)	0.53
FVC % post-BD, median (IQR)^4^	91 (80–102)	92 (83–103)	91 (79–101)	0.33
FEV_1_/FVC post-BD, median (IQR)^4^	80 (75–84)	80 (75–84)	80 (75–84)	0.93
DL_CO_ %, median (IQR)^5^	81 (70–92)	86 (77–95)	79 (67–89)	0.0001
MMRC score, median (IQR)^6^	0 (0–1)	0 (0–1)	1 (0–1)	0.038
SGRQ score, median (IQR)				
Total	7.1 (2.1–22.5)	4.1 (0.8–16.7)	8.6 (2.9–24.7)	0.0004
Symptom	11.1 (4.9–27.1)	10.2 (0.0–21.4)	12.9 (4.9–28.9)	0.047
Activity	12.2 (0.0–35.8)	6.2 (0.0–29.6)	12.5 (0.0–41.3)	0.007
Impact	1.9 (0.0–11.4)	0.0 (0.0–6.7)	3.6 (0.0–13.2)	0.003
6-MWD, mean; median m (IQR)	441;446 (388–493)	462;469 (418–512)	431;435 (376–481)	0.0001

PLWH: People living with HIV; IQR: interquartile range; Pack-year: median number packs of cigarettes smoked per day multiplied by number of years smoking; VL: HIV viral load; CD4: CD4 T-lymphocyte count; ART: antiretroviral therapy; SBP: systolic blood pressure; DBP: diastolic blood pressure; FEV1: forced expiratory volume during 1^st^ second; FVC: forced vital capacity; DL_CO_: diffusing capacity of the lungs for carbon monoxide adjusted for hemoglobin and carboxyhemoglobin; BD: bronchodilator test; % = percent-predicted; MMRC: Modified Medical Research Council dyspnea score; SGRQ: St George’s Respiratory Questionnaire; 6-MWD: Six minute walk distance

^1^n = 282

^2^n-284

^3^n = 297

^4^n = 422

^5^n = 425

^6^n = 316

* n = 295

P <0.003 are significant after adjusting for multiple comparison.

Post-bronchodilator percent-predicted forced expiratory volume in one second (FEV_1_)% predicted, forced vital capacity (FVC)% predicted and FEV_1_/FVC were similar between PLWH and HIV-uninfected individuals, but DL_CO_% predicted was significantly lower in PLWH (median 79 vs. 86, p = 0.0001). Average 6-MWD was significantly shorter for PLWH than for HIV-uninfected participants (mean 431 meters vs. 462 meters, p = 0.0001) and frequency of 6-MWD <350 meters in HIV-uninfected and PLWH was 5.4% vs. 16.2%, respectively (P = 0.002).

PLWH were also more likely to report respiratory symptoms as evidenced by the significantly worse SGRQ (median 8.6 vs 4.1, p = 0.0004) and MMRC scores (median 1.0 vs 0.0, p = 0.038). All three dimensions of SGRQ including symptom, activity and impact were significantly worse in PLWH (Table 1).

### Predictors of 6-MWD

Bivariate analysis: Lower FEV_1_%, FVC%, and DL_CO_% were associated with a shorter 6-MWD in both groups. Higher weight, MMRC and SGRQ score (where higher scores indicate greater symptom burden) were associated with shorter 6-MWD in both groups. In both groups, all three dimensions of SGRQ were significantly associated with lower 6-MWD. Spearman rho (P value) for symptoms, activity and impact score in PLWH was -0.13 (0.030), -0.25 (<0.0001), -0.26 (<0.0001). In the HIV-uninfected group, these correlations (P value) were -0.30 (0.0005), -0.40 (<0.0001), -0.34 (0.0001). Among PLWH, older age, Pittsburgh site and pack-year smoking were associated with shorter 6-MWD, whereas African-American race was associated with shorter 6-MWD in HIV-uninfected participants. Direction and/or magnitude of relationship between 6-MWD and age, pack-year smoking and site of recruitment were significantly different between the PLWH and -uninfected groups as reflected by the P value of interaction (Table 2).

**Table 2:** Pearson correlation (P value) of clinical and demographic variables with 6-MWD (m) in PLWH and HIV-uninfected participants. P value interaction shows the interaction between HIV status and each predictor to predict 6-MWD.

	HIV-uninfected	PLWH	P value for interaction
Age, year	0.0 (0.97)	-0.21 (0.0002)	0.037
Female	0.03 (0.73)*	-0.01 (0.92)*	0.53
Caucasian	0.29 (0.001)*	0.09 (0.10)*	0.13
Pitt Site	-0.08 (0.3)*	-0.37 (<0.0001)*	0.009
Height, cm	-0.07 (0.46)	0.01 (0.85)	0.50
Weight, kg	-0.29 (0.0007)	-0.18 (0.001)	0.37
Pack-year	-0.11 (0.22)	-0.29 (<0.0001)	0.032
Illicit drug usage	0.00 (>0.9)*	0.01 (0.88)*	0.72
Hemoglobin, g/dL	0.11 (0.24)	-0.01 (0.87)	0.31
VL, copies/ml	--	0.15 (0.012)	--
CD4, cell/μL	--	0.02 (0.69)	--
ART	--	-0.09 (0.13)	--
SBP, mmHg	-0.08 (0.35)	-0.05 (0.41)	0.77
DBP, mmHg	-0.06 (0.49)	-0.02 (0.76)	0.72
FEV1% post-BD	0.25 (0.004)	0.42 (<0.0001)	0.11
FVC% post-BD	0.20 (0.022)	0.42 (<0.0001)	0.06
FEV1/FVC post-BD	0.14 (0.10)	0.19 (0.001)	0.56
DL_CO_ %	0.21 (0.019)	0.31 (<0.0001)	0.30
MMRC score	-0.24 (0.005)	-0.27 (0.0002)	0.17
SGRQ score	-0.39 (<0.0001)*	-0.24 (<0.0001)*	0.95

6-MWD: Six minute walk distance; PLWH: People living with HIV; Pack-year: median number packs of cigarettes smoked per day multiplied by number of years smoking; VL: HIV viral load; CD4: CD4 T-lymphocyte count; ART: antiretroviral therapy use; SBP: systolic blood pressure; DBP: diastolic blood pressure;FEV1%: forced expiratory volume in one second; FVC%: forced vital capacity; BD: bronchodilator test; DL_CO_: diffusing capacity for carbon monoxide adjusted for hemoglobin and carboxyhemoglobin; % = percent-predicted; MMRC: Modified Medical Research Council dyspnea score; SGRQ: St George’s Respiratory Questionnaire;

* Spearman correlation (P value).

P <0.003 are significant after adjusting for multiple comparison.

### Multivariable analysis

In PLWH, DL_CO_% (β = 0.8), FEV1% (β = 0.5), age (β = -1.0 per year), height (β = 1.6 per cm), weight (β = -1.1 per Kg), pack-year smoking (β = -0.7), and SGRQ (β = -0.9) were independently associated with 6-MWD. White race was also associated with an average 25.6 meters greater 6-MWD. Both FVC% and FEV1% were independently associated with higher 6-MWD (β = 0.6). Each was entered the model separately given collinearity between the two values. CD4 count and HIV viral load were not associated with 6-MWD in PLWH. In contrast, DL_CO_% (β = 1.3), SGRQ (β = -1.7) and weight (β = -0.7) were the only independent predictors of 6-MWD in HIV-uninfected participants (Table 3). These models predicted 41% and 23% of 6-MWD variation in PLWH and HIV-uninfected participants, respectively.

**Table 3:** Predictors of 6-MWD (m) in PLWH and HIV-uninfected participants from multivariate regression analysis.

	PLWH*		HIV-uninfected **	
	Beta (95%CI)	P value	Beta (95%CI)	P value
Age, year	-1.02 (-1.8- -0.3)	0.009	NS	NS
Caucasian	25.6 (9.4–41.9)	0.002	NS	NS
Weight, kg	-1.1 (-1.6- -0.7)	<0.001	-0.7 (-1.4 - -0.005)	0.048
Height, cm	1.6 (0.7–2.5)	<0.001	NS	NS
Pack-years	-0.7 (-1.2- -0.3)	0.003	NS	NS
FEV1% post-BD ***	0.5 (0.01–1.0)	0.033	NS	NS
DL_CO_ %	0.6 (0.1–1.1)	0.005	1.3 (0.6–2.0)	0.001
SGRQ	-0.9 (-1.4- -0.4)	0.001	-1.7 (-2.4 - -0.9)	< 0.001
Constant	265.0 (95.9–434.2)	0.031	425.6 (343.8–507.5)	<0.001

*n = 289, three outliers were removed, R-square = 0.41

Model is adjusted for site.

**n = 127, three outliers were removed, R-square = 0.23

*** FVC% post could be replaced for FEV1% post with b = 0.6 (P = 0.035)

NS = Not statistically significant.

**Fig 1:**
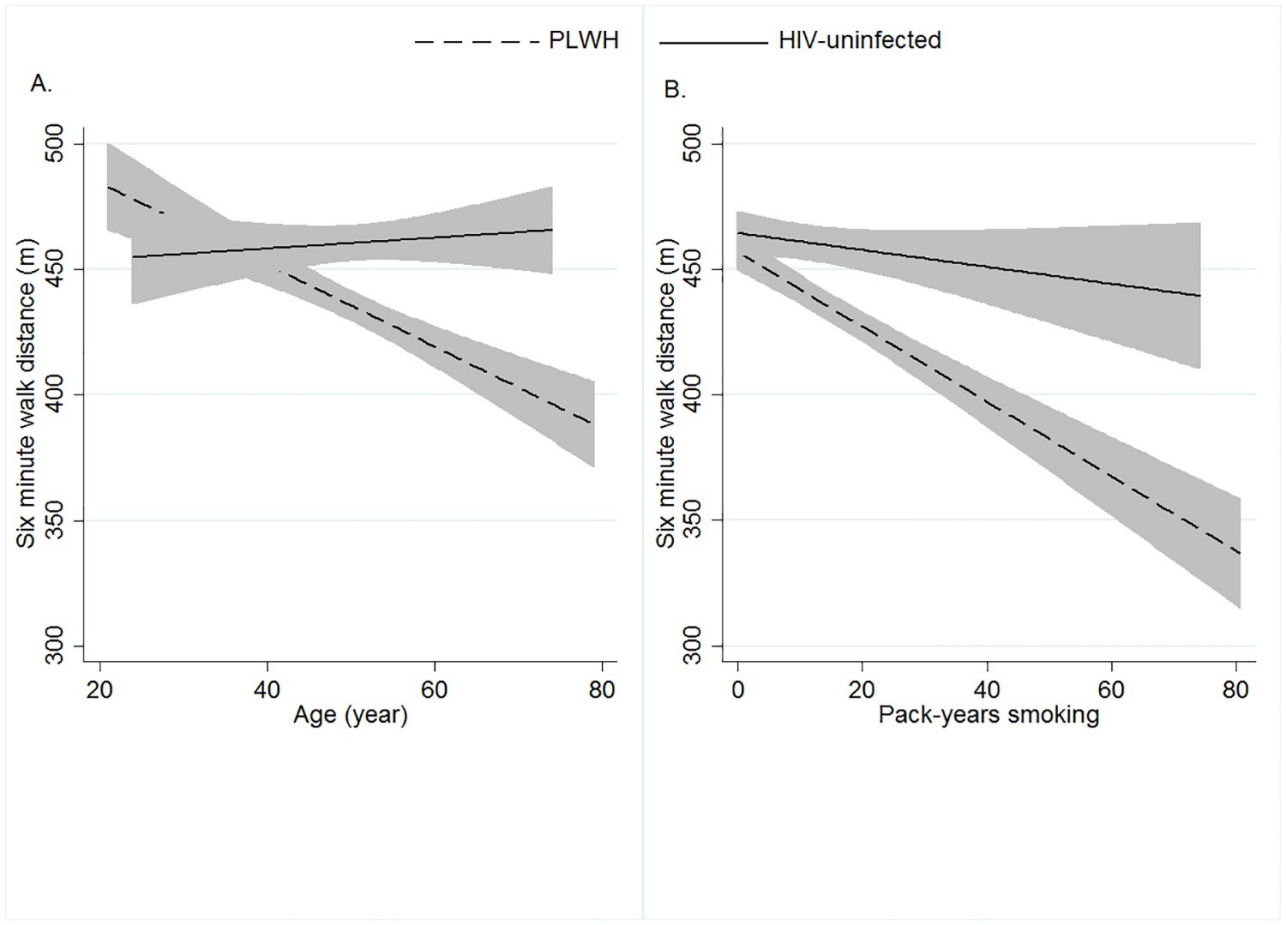
Relationship between 6-MWD, age and pack-year smoking. **(A)** relationship between age and 6-MWD in PLWH and HIV-uninfected individuals, P for interaction = 0.033 **(B)** relationship between pack-year smoking and 6-MWD in PLWH and HIV-uninfected individuals, P for interaction = 0.036. These figures show that higher pack-year smoking and older age decrease 6-MWD more in the PLWH compared to the HIV-uninfected group. Gray area represents 95%CI of regression line.

6-MWD: Six minute walk distance; PLWH: People living with HIV; Pack-year: median number packs of cigarettes smoked per day multiplied by number of years smoking; FEV1: forced expiratory volume in one second; % = percent-predicted; DL_CO_: diffusing capacity for carbon monoxide adjusted for hemoglobin and carboxyhemoglobin; SGRQ: St George’s Respiratory Questionnaire; FVC: forced vital capacity;

We then developed a pooled model including PLWH and HIV-uninfected individuals to test the adjusted effect of HIV on 6-MWD as well as the potential interaction of HIV and any significant predictors of 6-MWD. HIV had a significant, independent association with 6-MWD, with PLWH having an average 19.9 meters shorter 6-MWD (95% CI = -34.0- -5.9, P = 0.005) after adjusting for age, race, weight, height, smoking pack-years, SGRQ, DL_CO_% and FEV1%. Additionally, there were two significant interactions, one between pack-year smoking and HIV (beta = -0.9, P = 0.033, Fig 1a) and one between age and HIV (beta = -1.4, P = 0.036, Fig 1b) to predict 6-MWD.

## Discussion

We evaluated the relationships of HIV status, demographic and clinical variables, standardized respiratory questionnaires, and pulmonary function with 6-MWD in a cohort of PLWH and HIV-uninfected individuals. Although the majority of the cohort was virally-suppressed, HIV infection had an independent association with decreased 6-MWD, with average 6-MWD for PLWH approximately 20 meters less than for HIV-uninfected individuals when adjusted for covariates. We found that severity of airflow limitation and diffusing capacity impairment were independent predictors of decreased 6-MWD in PLWH, with only diffusing impairment being associated with 6-MWD in HIV-uninfected individuals. Worse SGRQ predicted shorter 6-MWD in both groups. HIV-associated variables such as CD4 cell count and ART use were not associated with 6-MWD.

There is conflicting evidence as to the independent relationship of HIV to physical function as measured by 6-MWD. During the ART era, two studies in primarily male PLWH and matched HIV-uninfected individuals did not show an independent effect of HIV on 6-MWD [9, 29]. A cross-sectional analysis of self-reported data from the Veterans Aging Cohort Study found very modest difference in physical function between PLWH and HIV-uninfected individuals. Interestingly, this study concluded that the presence of a co-morbid condition (chronic pulmonary disease) resulted in significant functional decline in PLWH relative to HIV-uninfected adults [5]. A study in older men found that all measures of physical performance including 6-MWD were significantly lower in PLWH when compared to a normal reference group [29]. PLWH in the Multicenter AIDS Cohort Study had a faster rate of decline in accelerated longitudinal gait speed over time when compared to HIV-uninfected men, but the associated clinical and demographic variables were not elucidated [30]. A recent study in middle-aged asymptomatic participants found subclinical deficits in physical function among PLWH [31]. Our study involved a more diverse population and revealed an independent effect of HIV on physical function. PLWH had shorter 6-MWD even after adjusting for potential clinical differences between PLWH and HIV-uninfected individuals. Differences from previous work likely result from differences in cohorts and measurements, as well as differences in ART regimens.

We found overlapping risk factors for decreased 6-MWD in PLWH and HIV-uninfected individuals, but also several unique predictors. First, DL_CO_ impairment was a common predictor of low 6-MWD in both groups. The relationship between DL_CO_ impairment and decreased 6-MWD is likely multifactorial, involving pulmonary vascular disease, airway obstruction, emphysema and inflammation [2]. Next, our findings of an independent relationship between SGRQ and 6-MWD are consistent with other studies, indicating that pulmonary symptom burden predicts lower walk distance [32, 33]. HIV infection and smoking share common pathways affecting pulmonary function, and PLWH may be more susceptible to the damaging effect of tobacco smoking [34]. Our finding that smoking (when overall burden of smoking in pack-years was relatively low) and impaired pulmonary function predict the 6-MWD only in PLWH suggests that at similar levels of spirometric dysfunction, PLWH have heightened susceptibility to functional impairment.

Other HIV-related factors such as CD4 cell count, HIV viral load, and use of ART were not related to 6-MWD, consistent with findings in previous studies [29]. In contrast, a prior study found that markers of HIV treatment success including higher CD4 counts and undetectable viral load were associated with improved physical function [35]. The high levels of well-controlled HIV within this cohort may have limited ability to assess the impact of these variables. Duration of HIV, nadir CD4 and zenith viral level may influence 6-MWD, but these data were not uniformly available in our cohort. Our findings do suggest that while the observed decline in physical function is related to HIV infection, it is likely independent of the level of immunosuppression.

It is possible that there are extra-pulmonary causes for reduced 6-MWD in PLWH. The associations between cardiac function, anemia and fatigue in HIV are well-known [3, 36]. Our study did not find an association between anemia and 6-MWD, likely due to the normal hemoglobin values among our study participants. We also did not systematically evaluate cardiovascular disease. Muscle wasting in the ART era is no longer a primary determinant of fitness [9]; however, aging, HIV infection and ART all have an additive effect on metabolism, pharmacokinetics, and immune function in PLWH which may contribute to declines in physical function [37]. A recent study indicated that PLWH have persistent inflammation, immune activation, and atypical skeletal muscle profiles that lead to asynchronous muscle aging [31]. Such effects of the virus may explain our finding that PLWH experience accelerated functional decline with age.

Underdiagnoses of co-morbidities is frequent in PLWH [8]. Our findings may have diagnostic and therapeutic implications by defining PLWH at higher risk of impaired physical function. The relationship of physical fitness to HIV-related variables is important, and by using performance-based measures of function, our study provides an initial assessment of the clinical and physiological determinants of cardiorespiratory fitness in PLWH when compared to an HIV-uninfected group.

The magnitude of differences observed between PLWH and uninfected participants is very similar to the minimum clinical important difference of 30.1 m [38] and has the potential to predict poorer health status, greater risk of disability, and higher morbidity. This finding emphasizes the importance of identifying modifiable risk factors for impaired physical performance including chronic airway obstruction in PLWH. Subjective assessment of respiratory symptoms, for example via SGRQ, may be a predictor of declining functional status and should prompt clinicians to implement strategies such as smoking cessation to improve functional capacity. Understanding factors that influence ambulatory function could have direct implications for guiding appropriate diagnostic evaluation of underlying disease processes in PLWH.

There are several limitations of our study. We used 6-MWD and not VO2, the gold standard for aerobic exercise capacity [39]. However, we believe that 6-MWD is a viable and readily-available surrogate as VO2 requires expensive equipment and trained staff, and the association between 6-MWD and VO2 peak has been found to be consistent [39]. We also did not assess for clinical pneumonia or colonization with respiratory organisms that have been shown to contribute to airway obstruction and could potentially affect 6-MWD [40]. We also had a smaller HIV-uninfected group that may have limited our power to see relationships between pulmonary function and 6-MWD. A larger, more diverse and longitudinal study of PLWH is also necessary for validation of impaired physical function as a prognostic indicator in this population.

Using a performance-based measure of function, we found that HIV infection was an independent predictor of decreased physical function and that impaired pulmonary function test and diffusing capacity abnormalities contribute to decreased 6-MWD. Pulmonary and/or cardiovascular disease in PLWH can confer an increased risk of morbidity making screening and early intervention important for this high-risk group. Impaired physical fitness, based on subjective or objective findings, may trigger clinicians to pursue further diagnostic workup, including assessing for underlying airflow obstruction or diffusion impairment. Research into clinical and molecular pathways leading to loss of physical function could help in understanding causes of functional impairment in this population.
